# Aminophylline shortage and current recommendations for reversal of vasodilator stress: an ASNC information statement endorsed by SCMR

**DOI:** 10.1186/s12968-018-0510-7

**Published:** 2018-12-20

**Authors:** Aiden Abidov, Vasken Dilsizian, Rami Doukky, W. Lane Duvall, Christopher Dyke, Michael D. Elliott, Fadi G. Hage, Milena J. Henzlova, Nils P. Johnson, Ronald G. Schwartz, Gregory S. Thomas, Andrew J. Einstein

**Affiliations:** 1 Wayne State University and John D. Dingell VA Medical Center, Detroit, MI USA; 2 University of Maryland, Baltimore, MD USA; 3 Cook County Health, Chicago, IL USA; 4 Hartford Hospital, Hartford, CT USA; 5 Alaska Heart and Vascular Institute, Anchorage, AK USA; 6 Carolinas Medical Center, Charlotte, NC USA; 7 University of Alabama at Birmingham and Birmingham VA Medical Center, Birmingham, AL USA; 8 Friendship Center, Sarasota, FL USA; 9 University of Texas Medical School, Houston, TX USA; 10 University of Rochester, Rochester, NY USA; 11 MemorialCare Heart & Vascular Institute, University of California, Irvine, Long Beach, CA USA; 12 Department of Medicine, and Department of Radiology, Columbia University Irving Medical Center and New York-Presbyterian Hospital, 622 West 168th Street PH 10-203, New York, NY 10032 USA

## Abstract

Pharmacologic reversal of serious or intolerable side effects (SISE) from vasodilator stress is an important safety and comfort measure for patients experiencing such effects. While typically performed using intravenous aminophylline, recurrent shortages of this agent have led to a greater need to limit its use and consider alternative agents. This information statement provides background and recommendations addressing indications for vasodilator reversal, timing of a reversal agent, incidence of observed SISE with vasodilator stress, clinical and logistical considerations for aminophylline-based reversal, and alternative non-aminophylline based reversal protocols.

## Overview

The purpose of this document is to provide cardiac imaging specialists performing vasodilator stress testing with specific recommendations regarding options for the reversal of vasodilator stress. The timeliness of this document is associated with recurrent shortages of aminophylline, which has been used in most laboratories for reversal of serious and intolerable side effects (SISE) from vasodilator stress.

This information serves as a summary of expert opinion on this topic developed by the American Society of Nuclear Cardiology (ASNC) and endorsed by the Society for Cardiovascular Magnetic Resonance (SCMR). This document covers general indications for reversal of SISE from vasodilator stress, the standard reversal procedure using intravenous (IV) aminophylline, and alternative options based on evidence and expert opinion for effective and safe reversal of SISE from vasodilator stress during pharmacologic stress myocardial perfusion imaging (MPI) with single photon emission computed tomography (SPECT), positron emission tomography (PET), or cardiovascular magnetic resonance (CMR).

## Indications for vasodilator stress reversal

ASNC 2016 imaging guidelines for SPECT nuclear cardiology procedures[1] support using aminophylline for reversing the effects of vasodilator stress testing with adenosine, regadenoson and dipyridamole when SISE are encountered. The guidelines reflect evidence that most vasodilator stress-related adverse effects are self-limiting and do not require reversal. Serious adverse effects can be effectively aborted with IV administration of aminophylline, a xanthine derivative and a nonselective adenosine receptor antagonist [2], with the exception of seizures. Intravenous lorazepam should be the first-line intervention for seizures and methylxanthine use is not recommended for reversal of adenosine or regadenoson effects under these circumstances because of the concern for possible lowering of the seizure threshold and potential risk of exacerbating seizures [3], based on limited evidence and the preponderance of expert opinion.

Based on the 2016 guidelines[1], indications for reversal of vasodilator stress using IV aminophylline (50 to 250 mg intravenously at least 1 min after the tracer injection) include the following:

1. Severe hypotension (systolic blood pressure (BP) ≤80 mmHg) or symptomatic hypotension;
2. Development of symptomatic, persistent Mobitz II second degree or complete heart block;
3. Significant cardiac arrhythmia (e.g. ventricular tachycardia);
4. Wheezing;
5. Severe chest pain associated with ST depression of 2 mm or greater, or ST elevation of 1 mm or greater;
6. Signs of poor perfusion (pallor, cyanosis, cold or clammy skin);
7. Intolerable symptoms such as nausea, vomiting, or abdominal pain.

It is important for clinicians to identify patients at risk for SISE who may need prompt reversal of vasodilator stress agent. Those at risk include patients with borderline low systolic BP (< 100 mmHg), baseline 1st or 2nd degree atrioventricular block, reactive airway disease, or acute coronary syndrome presentation [4]. Patients with end-stage renal disease tend to have higher frequency of regadenoson-induced gastrointestinal side effects [5, 6].

For CMR stress with regadenoson, in addition to SISE, pharmacologic reversal may be required for a minority of examinations (< 25%) in which rest perfusion imaging is needed for artifact differentiation observed on stress imaging. In such a case, given the half-life of regadenoson and the short window between stress and rest CMR perfusion imaging, vasodilator stress reversal may be deemed necessary if there is concern for residual drug effect.

## Timing of a reversal agent

Few studies have examined how early following radioisotope administration a reversal agent for vasodilator stress can be administered without influencing the results of perfusion imaging.

The majority of myocardial uptake of the radioisotope occurs within 1–2 min following the radioisotope injection [7–10]. Thus, it is important to maintain vasodilator-induced myocardial hyperemic state during this period. However, if a very serious adverse event were to occur, the vasodilator should be reversed immediately. If vasodilator agent reversal occurs in the first minute following radioisotope injection, the “stress” perfusion could well be compromised. Therefore, following a premature reversal of vasodilator stress, if myocardial perfusion imaging is normal or myocardial ischemia is absent in a patient with high likelihood of ischemic heart disease, the possibility of a “false-negative” scan should be considered and further testing with an alternative stress agent (such as dobutamine) should be considered. For stress CMR, a reversal agent can be given once first-pass stress perfusion images have been acquired.

## Incidence of observed side effects with vasodilator stress

Based on published evidence, the vasodilator stressor agents used in current cardiology practice are safe and have a very low observed rate of serious adverse effects (Table 1). Mild, easily reversible adverse effects are frequent. Perceived shortness of breath associated with regadenoson is commonly seen and does not reflect elevated pulmonary capillary wedge pressure, but may reflect a central nervous system effect [11]. Gastrointestinal side effects are overall mild/transient and are more frequently observed in patients with end-stage renal failure undergoing regadenoson stress [5, 6].

**Table 1:** Adverse effects of the vasodilator stressor agents (data derived from 2016 ASNC imaging guidelines for SPECT nuclear cardiology procedures [1], from Subbiah et al. [19], and from Doukky et al. [20])

	Dipyridamole	Adenosine	Regadenoson
Minor side effects (total)	~ 50%	~ 80%	~ 50%
flushing	3%	35–40%	17%
nonspecific chest pain	20%	25–30%	11%
dyspnea	10%	20%	25%
dizziness	12%	7%	7%
hypotension	5%	5%	5%
nausea	5%	5%	6%
headache	12%	10%	29%
abdominal discomfort	5–10%	5–14%	6–17%
Transient conduction abnormalities:			
Overall	2%	8.5%	4%
Transient 2nd degree AVB	1–2%	4%	0.1%
Transient ST depression of ≥1 mm	8%	5–7%	1–2% [20]
Major side effects	< 1%	< 1%	< 1%
Complete AVB	< 1%	< 1%	Rare
Severe (> 2 mm) ST depression	< 1%	< 1%	< 1%
Fatal or nonfatal MI	Extremely rare	Extremely rare	Extremely rare
Stroke	Extremely rare	Extremely rare	Extremely rare
Seizures	Rare	Rare	Rare
Comments	Symptoms may last longer (15–25 min) than with other vasodilator stressors; Aminophylline frequently required	Due to a short half-time, most side effects resolve in a few (< 10) seconds after discontinuation of the infusion; Aminophylline rarely required	Most adverse reactions begin soon after injection and resolve within 15 min (headaches may last up to 30 min)

*AVB* atrioventricular block; *MI* myocardial infarction

Table 2 [3] provides more detailed information regarding SISE associated with vasodilator stress agents which essentially represents a list of indications for the reversal procedure (with the exception of seizures). As can be seen from these data, the rate of occurrence of clinical situations matching the list of indications for reversing vasodilator stress is very infrequent.

**Table 2:** Serious and fatal complications of exercise and pharmacologic cardiac stress testing (rate of observed and reported events per 1000). Reproduced with permission from Dilsizian et al. [3]

	Exercise	Dobutamine	Dipyridamole MPI	Adenosine MPI	Regadenoson MPI	Gadolinium MPI
Any serious complication	0.1–3.46	2.988	0.714–2.6	0.97	Case reports	NR
Death	0–0.25	Case reports	0.5	Case reports	Case reports	NR
VFib/VTach	0–25.7	0.6–1.35	NR	NR	NR	NR
Acute MI	0.038	0.3–3.0	1.0	0.108	Case reports	NR
Cardiac rupture	Unknown	Case reports	NR	NR	NR	NR
High degree AVB or asystole	Unknown	NR	Case reports	Case reports	Case reports	NR
Bronchospasm	Unknown	NR	1.5	0.76	Case reports	NR
Stroke/TIA	Unknown	Case reports	NR	NR	Case reports	NR
AFib	Unknown	5–40	NR	NR	Case reports	NR
Seizure	Unknown	Case reports	NR	1.5	Case reports	NR
CIN	NR	NR	NR	NR	NR	NR
NSF	NR	NR	NR	NR	NR	0–18%^a^
Radiation-Induced Cancer	NR	NR	NR (theoretical)	NR (theoretical)	NR (theoretical)	NR

*AVB* atrioventricular block, *AFib* atrial fibrillation, *CIN* contrast induced nephropathy, *MI* myocardial infarction, *NSF* nephrogenic systemic fibrosis, *NR* not reported, *TIA* transient ischemic attack, *VFib* ventricular fibrillation, *VTach* ventricular tachycardia

^a^Rate of NSF is negligible if not zero in the current era of not administering gadolinium if the estimated glomerular filtration rate is < 30 ml/min/1.74 m^2^

References for entries are reported in Dilsizian et al. [3]

Considering combined evidence of serious adverse effects of clinically utilized vasodilator agents and actual guidelines-endorsed indications for aminophylline use, a nuclear cardiology or CMR laboratory will encounter occasional cases that require vasodilator stress reversal. The rate of these cases has not been definitively assessed but it is certainly less than 10% and may be as low as 1% based on Tables 1 and 2. With periodic shortage of aminophylline, selective use of reversal agents is a reasonable alternative to routine use and does not compromise safety or effectiveness of testing.

## Aminophylline-based vasodilator stress reversal: Clinical and logistical considerations

Despite the data demonstrating that only a fraction of vasodilator stress studies require a reversal procedure, present day cardiac imaging laboratories utilize IV aminophylline much more frequently and with high regional variability. In different clinical practices in the US, the proportion of vasodilator stress tests with aminophylline use ranges from less than 3 to 100% [12], the latter being in an attempt to prevent any possible adverse effects of the vasodilator stress.

Randomized controlled trials demonstrate use of aminophylline is safe, well tolerated, and effective in improving overall patient satisfaction with vasodilator stress testing, the latter by reducing uncomfortable and easily treatable symptoms [13, 14]. In pooled analysis of two randomized but non-crossover trials including 548 patients, investigators found that perfusion did not appear to be impacted if aminophylline was administered 1.5 min following Tc-99 m tetrofosmin injection [15]. Until further studies are performed, a delay of 2 or more minutes after tracer injection is prudent if reversal is indicated. With this delay, if SISE are encountered during vasodilator stress, the reversal procedure should be performed without concern for altering imaging findings.

To date, we do not have any published evidence whether routine or “prophylactic” vasodilator stress reversal using IV aminophylline (or any other reversal agent), regardless of whether or not a patient develops an adverse reaction, is clinically beneficial. Such prophylactic use is not included in the package inserts of regadenoson, adenosine, or dipyridamole. However, it may unnecessarily complicate the performance of vasodilator stress, introduce an intervention that may affect imaging findings if administered too soon after tracer injection, and, at least hypothetically, increase risk due to exposure to an unnecessary medical agent. Accordingly, this approach is not recommended.

## Alternative non-aminophylline based vasodilator stress reversal protocols

When aminophylline is in short supply but reversal of SISE from vasodilator stress is indicated, alternative reversal agents that have been shown to be effective in this setting should be used.

One such agent is another xanthine derivative, theophylline, which has been reported to reverse adverse symptoms of the vasodilator stressor agents safely and effectively. Since aminophylline is a salt that contains approximately 80% theophylline by weight, lower doses of theophylline can achieve the same reversal effects. Moreover, due to differences in concentration between aminophylline (25 mg/mL) and theophylline (0.8 mg/mL), it becomes impractical to administer more than 50 mg of theophylline as a single dose, equivalent to roughly 60 mg of aminophylline. A total of 5 published series with 215 patients support the use of IV theophylline as a vasodilator stress reversal agent [16]. In the largest such series, 154 patients received theophylline for reversal of dipyridamole; only 11 patients (7%) required a second injection, and in all cases side effects or frank ischemia from dipyridamole were successfully treated [16].

Another widely available methylxanthine inhibitor of the adenosine receptors (including adenosine 2A receptors activated by regadenoson) is caffeine. This agent is administered via either an IV or oral route. Both oral and IV caffeine were recently compared in a randomized controlled fashion to IV aminophylline by Doran et al.[17] and were shown to provide rapid and safe reversal of vasodilator-induced adverse effects during SPECT MPI. This study evaluated the effect of reversal agents on patient-reported symptoms in a series of 241 consecutive patients presenting to the stress laboratory and undergoing regadenoson stress. Patients were randomized to IV aminophylline, to 60 mg of IV caffeine over 3 to 5 min, or to oral caffeine as either regular coffee or diet cola taken ad lib (estimated 60–160 mg oral caffeine, with no exact dose available). No serious adverse effects of regadenoson were encountered. Of the 241 patients undergoing regadenoson stress, 152 (63%) received a reversal agent. Rates of complete or predominant reversal were 100% with IV aminophylline, 96% with IV caffeine (P=NS vs aminophylline), and 84% with PO caffeine (*P* = 0.003 vs IV aminophylline). Of the 37 patients in the oral caffeine group, 19 assigned to oral caffeine crossed over to receive IV caffeine based on concern the symptoms might be too severe for oral caffeine to be effective [17].

Alternative methods of caffeine administration have also been used, such as buccal administration, which is thought to be rapid and well tolerated. The only letter addressing this delivery method[18] did not report rates of successful reversal of side effects or ischemic electrocardiographic changes, or the need for additional pharmacologic reversal, and thus this approach still warrants formal investigation. Direct IV injection of caffeine over a shorter period of 30 to 60 s has not been studied to date.

Data regarding use of the alternative reversal agents is summarized in Table 3. Of note, calculation of the IV or oral caffeine dose comparable with an index dose of IV aminophylline is challenging and not always feasible [12].

**Table 3:** Aminophylline and Alternative Vasodilator stress reversal agents

Reversal Agent	HCPCS Code	Formulation	Cost per unit dose^a^	Recommended dose	Additional recommendations
IV Aminophylline	J0280	250 mg in 10 mL vial	$9.92	50–125 mg; slow manual injection	May repeat additional 50–125 mg as needed if the initial dose has not completely eliminated symptoms
IV Theophylline	J2810	400 mg in 500 mL bag	$3.50	50 mg; slow manual injection (over 1 min)	May repeat additional 50 mg if needed
IV Caffeine Citrate	J0706	60 mg in 3 mL vial	$6.05	60 mg diluted in 25 cc D5W; infused over 3–5 min	May repeat an infusion (30–60 mg) if needed^b^
PO Caffeine	–	Caffeinated beverage	–	Estimated 60–160 mg; given as a regular coffee, tea or caffeinated beverage^c^	Consider switching to IV Aminophylline or IV Caffeine if lack of complete resolution of symptoms achieved

HCPCS code The Healthcare Common Procedure Coding System (HCPCS) is a collection of codes that represent procedures, supplies, products and services which may be provided to Medicare beneficiaries and to individuals enrolled in private health insurance programs. *IV* intravenous; *mg* milligram; *PO* oral; ^a^Cost data provided by Pete Antonopoulos, PharmD, Cook County Health, October 15, 2018. ^b^No definitive evidence available regarding the repeat dose. ^c^Most regular coffees contain over 100 mg of caffeine; see data from Center for Science in the Public Interest data in Appendix 1 of 2016 ASNC Stress Protocols and Tracers guidelines.[1] For example, a 16 fluid ounce Starbucks Grande has 330 mg, and a Keurig Coffee K-Cup which makes 8 oz of coffee has 75–150 mg. A 12 oz Diet Coke has 47 mg; the Food and Drug Administration limit for cola and pepper soft drinks is 71 mg per 12 oz.

Finally, adding low-level exercise to vasodilator stress in patients undergoing MPI (except patients with left bundle branch block or right ventricular pacemaker), may minimize some adverse reactions during and after the vasodilator stress, but cannot completely eliminate these negative effects [17]. Thus, reversal agents should be available in any stress laboratory performing vasodilator stress testing and medical staff should be familiar and efficient in recognizing SISE.

While reversal of the adverse effects is an important part of safety protocols in the modern nuclear cardiology or cardiac magnetic resonance laboratory, vasodilator induced coronary steal and ischemia in patients with coronary disease may produce angina, ischemic electrocardiographic changes and/or ischemic arrhythmias. Therefore, approved advanced cardiac life support (ACLS) protocols, in addition to reversal agent administration, must be readily available. Similar to ischemic complications, non-cardiac severe adverse reactions, such as severe wheezing, headache or seizures, require availability of a full spectrum of emergent therapeutic measures targeting these symptoms.

In the absence of comparative effectiveness studies, given the chemical similarities (aminophylline is a mixture of theophylline and ethylenediamine), the panel recommends the use of IV theophylline as a first choice reversal agent where possible when aminophylline is not available. However, shortages of these drugs have been noted in tandem, so when neither is available, IV caffeine should be used for reversal of SISE.

## Summary

Vasodilator stress related adverse reactions and complications may require implementation of a reversal procedure in approximately 1–10% of patients. There is no definitive published evidence showing any significant clinical benefit of the preventive use of vasodilator stress reversal in patients undergoing vasodilator stress MPI who are asymptomatic. Patients should be routinely advised prior to testing that rapid, safe and effective reversal of any adverse effects of vasodilator stress testing is immediately available. Based on the available evidence, several effective and safe options for reversal of vasodilator stress-related complications/adverse reactions are available. These include IV aminophylline, theophylline, and caffeine. Theophylline and caffeine can be substituted for aminophylline in the nuclear cardiology or cardiac magnetic resonance practice in the event of a shortage of IV aminophylline. Moreover, oral caffeine is typically effective in mild cases of vasodilator stress-associated adverse reactions for most patients. These reversal agents have been shown to be safe and effective.

## Abbreviations

ACLS: advanced cardiac life support
ASNC: American Society of Nuclear Cardiology
CMR: cardiovascular magnetic resonance
IV: intravenous
MPI: myocardial perfusion imaging
PET: positron emission tomography
SCMR: Society for Cardiovascular Magnetic Resonance
SISE: serious and intolerable side effects
SPECT: single photon emission computed tomography
